# Raman microspectroscopy fingerprinting of organoid differentiation state

**DOI:** 10.1186/s11658-022-00347-3

**Published:** 2022-06-28

**Authors:** Kate Tubbesing, Nicholas Moskwa, Ting Chean Khoo, Deirdre A. Nelson, Anna Sharikova, Yunlong Feng, Melinda Larsen, Alexander Khmaladze

**Affiliations:** 1 Department of Physics, University at Albany, State University of New York, 1400 Washington Avenue, Albany, NY 12222 USA; 2 Department of Biological Sciences, University at Albany, State University of New York, 1400 Washington Avenue, Albany, NY 12222 USA; 3 RNA Institute, University at Albany, State University of New York, 1400 Washington Avenue, Albany, NY 12222 USA; 4 Department of Mathematics, University at Albany, State University of New York, 1400 Washington Avenue, Albany, NY 12222 USA; 5 Present Address: The Jackson Laboratory, 10 Discovery Dr., Farmington, CT 06032 USA; 6 Present Address: Neural Stem Cell Institute, Rensselaer, NY 12144 USA

**Keywords:** Raman spectroscopy, Tissue-engineered organoids, Salivary gland organoids, Regenerative medicine

## Abstract

**Background:**

Organoids, which are organs grown in a dish from stem or progenitor cells, model the structure and function of organs and can be used to define molecular events during organ formation, model human disease, assess drug responses, and perform grafting in vivo for regenerative medicine approaches. For therapeutic applications, there is a need for nondestructive methods to identify the differentiation state of unlabeled organoids in response to treatment with growth factors or pharmacologicals.

**Methods:**

Using complex 3D submandibular salivary gland organoids developed from embryonic progenitor cells, which respond to EGF by proliferating and FGF2 by undergoing branching morphogenesis and proacinar differentiation, we developed Raman confocal microspectroscopy methods to define Raman signatures for each of these organoid states using both fixed and live organoids.

**Results:**

Three separate quantitative comparisons, Raman spectral features, multivariate analysis, and machine learning, classified distinct organoid differentiation signatures and revealed that the Raman spectral signatures were predictive of organoid phenotype.

**Conclusions:**

As the organoids were unlabeled, intact, and hydrated at the time of imaging, Raman spectral fingerprints can be used to noninvasively distinguish between different organoid phenotypes for future applications in disease modeling, drug screening, and regenerative medicine.

**Supplementary Information:**

The online version contains supplementary material available at 10.1186/s11658-022-00347-3.

## Highlights

- Salivary gland organoids have unique Raman signatures detectable with a confocal-based Raman imaging approach.
- Raman signatures can be detected in unlabeled fixed or live organoids.
- Raman spectral signatures effectively predict organoid phenotypes.

**Supplementary Information:**

The online version contains supplementary material available at 10.1186/s11658-022-00347-3.

## Background

Organoids are essential biological tools, utilized across multiple fields, as they represent the organization and function of multiple cell types in 3D and often provide translationally valuable insight into drug responses, disease pathology, and developmental biology [1]. There are various protocols to produce organoids. However, as all of them are labor-, material-, and time-intensive, better approaches are required to improve workflow and cost. Organoids can be created from the culture of either stem cells or progenitor cells within an extracellular 3D matrix environment. With the correct combination of cell types and medium components, the cells self-organize and differentiate to form 3D cell structures with distinct organ-like characteristics [1, 2]. However, organoids are inherently heterogeneous [3]. For applications such as disease modeling and drug screening, where monitoring an organoid’s differentiation state over time is critical, there is a need for noninvasive imaging methods. Organoids that express fluorescent or luciferase proteins have been used in numerous screening applications [4–6]. However, the use of exogenous protein expression systems and labels is limiting and is not well suited for most preclinical applications. Therefore, there is a need for nondestructive methods that confirm developmental or phenotypic stages in both live and fixed organoids, which are intact, hydrated, and unlabeled.

Raman spectroscopy offers a nondestructive, label-free approach for classifying biological samples [7]. The Raman effect is a natural phenomenon of inelastic light scattering determined by the vibrational energy levels of specific molecular structures [8]. There are numerous variations on traditional Raman approaches; many require labeling of samples with a Raman-sensitive compound [9, 10]. As labels may change their state, for monitoring of organoids, unlabeled samples are ideal. The interpretation of the Raman spectra of biological samples is often dependent on the spectral resolution of the method, with numerous peaks assigned to biological components such as DNA, RNA, protein, or lipids. These peak assignments are based on prior measurements [7]. Often the “Raman signature” or spectral trends, rather than individual peaks, are utilized to distinguish between biological conditions. Although Raman spectroscopy is frequently compared to Fourier transform infrared (FTIR) spectroscopy, Raman spectroscopy is superior for biological samples, as it does not suffer from water interference as FTIR does [11]. In both surgical and clinical pathology settings, low-spatial-resolution Raman measurements can detect large structural differences in tissues, including the presence of fibrillar collagens, or discriminate healthy tissue regions from disease- or tumor-burdened regions [12–18]. With the scale determined by the numerical aperture of the microscope objective and the confocal settings of the instrument, high-resolution Raman microspectroscopy can be used to gain comprehensive information on the chemical composition of subcellular or cellular populations [19–23].

Here, we developed a Raman method to classify live salivary gland organoids. We used a well-characterized salivary gland organoid model that is responsive to signaling by fibroblast growth factor 2 (FGF2) and epidermal growth factor (EGF) [2, 24, 25]. We investigated the use of Raman spectroscopy to noninvasively recognize and predict the organoids’ differentiation state. Our developed methods minimize background extracellular matrix (ECM) signals and use Raman confocal microspectroscopy to define specific spectral signatures for organoids in different differentiation states. Quantitative methods and machine learning were used to classify distinct organoid differentiation signatures and reveal Raman spectral signatures or “fingerprints” that are predictive of organoid phenotype. This approach is promising for numerous translational and preclinical organoid models that cannot utilize fluorescent or other labels to probe their differentiation state.

## Materials and methods

### Mouse submandibular gland cell isolation

Embryonic day 16 (E16) timed-pregnant CD-1 female mice were ordered from Charles River Laboratories. Submandibular glands (SMGs) were removed from E16 embryos following protocols approved by the University at Albany Institutional Animal Care and Use Committee (IACUC). SMG removal involved slicing the mandible with a scalpel and then removing the glands using sterile forceps under a dissecting microscope. Epithelial and mesenchymal cell populations were enriched, as previously described and outlined briefly here [2, 24]. SMGs were microdissected in 1× phosphate-buffered saline (PBS) containing collagenase/hyaluronidase (StemCell Technologies, #7912) and dispase II (Life Technologies, #17105041), followed by manual trituration, and epithelial cell populations were enriched by gravity sedimentation. The mesenchymal-enriched cell population in the supernatant was further enriched by filtration through 70-µm (Falcon #087712) and 40-µm cell strainers (Fischer Scientific #22363547), washed by pelleting at 450*g* for 5 min, and resuspended in medium, Dulbecco’s Modified Eagle Medium/Nutrient Mixture F12 (DMEMF12, Fisher #21041025) containing 5000 units/mL of penicillin and 5000 µg/mL of streptomycin (Pen-strep, Fisher #15070063) and 10% fetal bovine serum (Life Technologies #10082147). All animal work was performed in compliance with the PHS Policy on Humane Care and Use of Laboratory Animals, as mandated by the Health Research Extension Act of 1985 and the USDA Animal Welfare Regulations, and was approved by the UAlbany IACUC committee: IACUC protocols #19-003 and 22-002.

### Epithelial organoid formation

About 900 epithelial clusters (1.0 gland equivalent) with a 20,000–50,000 stromal/mesenchymal cell addition (0.2 gland equivalent) were embedded in Matrigel (Corning #356234) at a 1:1 cell to Matrigel ratio. Ten microliters of the cell and basement membrane mixture was seeded into the well of a 50 mm glass-bottom dish (MatTek #P50G-1.5-14F). The Matrigel was solidified by incubating at 37 °C in a tissue culture incubator (Thermofisher Scientific Forma Series II) for 15 min and covered with 180 µL of DMEM/F-12/10% FBS/Pen-Strep with or without growth factors added. The growth factor concentrations used were 100 ng/ml epidermal growth factor (EGF) (PeproTech #AF100-15) or fibroblast growth factor-2 (FGF2) (Peprotech #450-33) solubilized in 0.2% BSA and stored at −20 °C in single-use aliquots. Organoids were cultured for 7 days at 37 °C in a tissue culture incubator in 5% CO_2_ with the medium replaced once at day 4. After 7 days of culturing, organoids were fixed by replacing the medium with 4% paraformaldehyde (PFA) (Electron Microscopy Sciences #15710) in 1× PBS for 20 min and stored in 1× PBS prior to Raman imaging or immunocytochemistry.

### Immunocytochemistry, fluorescent imaging, and analysis

Immunocytochemistry (ICC) was performed, as described previously [26, 27], with 0.4% Triton-X 100 (Sigma #T9284-100ML) used for permeabilization of 4% PFA-fixed samples. All primary antibody incubations were overnight at 4 °C. Secondary antibodies were incubated for 1–3 h at room temperature. Primary antibodies and dilutions used included aquaporin 5 (AQP5, 1:400; Alomone #AQP-005), cytokeratin 7 (K7, 1:200; Abcam #ab9021), and epithelial cell adhesion molecule (EpCAM) directly conjugated to fluorescein isothiocyanate (FITC) (1:400; eBiosciences #11-5791-82). Secondary antibodies, including cyanine and Alexa dye-conjugated AffiniPure F(ab’)2 fragments, were purchased from Jackson ImmunoResearch Laboratories and used at a dilution of 1:250. For nuclei staining, 4′,6-diamidino-2-phenylindole (DAPI) (Life Technologies #D1306) was used in conjunction with secondary antibodies. Mounting medium contained 90% (vol/vol) glycerol (Sigma #G5516-1L) in 1× PBS with 1–4 diazobicyclo[2,2,2]octane (DABCO) (Sigma #D27802-100G) and *n*-propyl gallate (Sigma #P3130-100G) as an antifade agent. Imaging was performed using a Zeiss Z1 Cell Observer wide-field microscope (10× or 20×) or Zeiss LSM 710 confocal microscope (63×, oil immersion) with the same configuration for all samples within an experiment. Quantification of immunostained area and organoid sizes was performed using FIJI v1.53c and postprocessed with rolling-ball background subtraction and line tool [28]. All statistical significance calculations for ICC were performed using a single-factor ANOVA followed with post-hoc Tukey HSD test for multisample comparison. Statistical tests on ICC data were performed in Microsoft Excel (Microsoft Corporation) or R (R Core Team).

### Raman microspectroscopy collection and processing

Prior to Raman imaging, fixed organoids were transferred to an imaging chamber (Warner) filled with 1× PBS and with a quartz coverslip bottom (Esco Optics). There organoids were inspected, and brightfield images collected on AmScope with a 4× objective. Live organoids remained in glass-bottom dishes, with phenol red-free medium for Raman imaging. Imaging time was reduced to ensure the samples spent less than 20 min outside of a 37 °C incubator. All Raman spectra were collected using a Horiba XploRA Plus confocal Raman microscope with a built-in 1024 × 256 TE air-cooled Syncerity CCD camera (pixel size 26 μm, temperature −60 °C). A second camera, incorporated into the Horiba system, was employed for the collection of brightfield images of the samples on the stage. All Raman measurements of the organoids were made with a plan N 4× Olympus objective [numerical aperture (NA): 0.10, infinity corrected], 532 nm laser (100% full power), 1800 g/mm grating. For fixed organoids, we used 500–2000 cm^−1^ spectral region, 100 µm slit, 300 µm confocal aperture, 30 s acquisition time, and three accumulations per point. To reduce imaging time, the live organoids were measured with a 600–2000 cm^−1^ spectral region, 100 µm slit, 500 µm confocal aperture, 15 s acquisition time, and three accumulations per point. In both live and fixed organoid imaging, the accumulations per point were averaged by the program with an integrated spike removal feature during the active collection process. Random spikes, which were not identified by LabSpec6 software, were removed manually if 3.5× higher than the average signal; the gap was filled with the average value of the two connecting spectral points.

To identify the cell-dense regions within Matrigel, brightfield images of organoids were collected for determination of their *X*–*Y* coordinates, which was followed by point-by-point scans with multiple *Z* depths. Raman spectra collection from control, EGF-, and FGF2-treated salivary organoids focused on the cell-dense regions, with processing to eliminate Matrigel-rich regions. Sampling of fixed organoids included five locations scanned per image, with three to four different *Z* depths (step of 150 µm). Live organoid sampling increased sampling locations and decreased *Z*-depth evaluation to reduce imaging time. An average of 40–60 spectra were collected from three organoids per treatment within a single experiment, with three replicate experiments performed; representative experiments are shown. Spectra representing Matrigel-rich regions, which were identified by saturation in fixed organoids or a significantly increased background in live organoids, were removed from further analysis. Spectral processing was performed using the HORIBA LabSpec6 software, which included de-noise (first-degree polynomial method with size 4), fluorescent background removal (second-degree polynomial fit with 256 points for fixed samples and fifth degree and 100 points for live samples), with normalization to the phenylalanine peak (~ 1003 cm^−1^).

### Spectral analysis methods: peak ratio quantification, singular value decomposition (SVD), and machine learning

Peak ratio analysis was completed with a normalized spectral dataset in a spreadsheet (Excel, Microsoft). Statistical tests for peak ratio analysis included single-factor ANOVA with a Dunnett’s post hoc and Student’s *t*-test with Bonferroni correction (statistical values provided in Additional file 1: Tables). In-house MATLAB code was used to implement the SVD analysis [19]. The MATLAB code took stacked Raman spectra as the input *n* × *m* matrix, where *n* is the number of points in the spectrum and *m* is the number of Raman spectra in the dataset. The input matrix was decomposed into matrices U, Σ, and V^T^, where matrix V was used to generate SVD scatter plots, while individual SVD components were collected in matrix U.

The machine learning analysis was performed with robust support-vector machines (RSVM), which has been previously described [29], and visualized with violin plots [30]. Each comparison was made by splitting data into a training set (83–90% spectra) and classification test set (10–17% total spectra). The RSVM approach was run 4000 times per comparison. For all experiments, the standard deviation of the classification accuracy is below 0.10. The supporting software for RSVM analysis is MATLAB.

### Blinded evaluation of organoid spectra with peak ratio and SVD evaluation

A total of ten spectra was excluded from each control, EGF, and FGF2 dataset and renamed unknown 1–3. The average peak intensity of unknown groups was acquired blind at 569, 621, 676, 1124, 1248, 1335, 1446, 1654, and 1927 cm^−1^ and compared with the known sample mean peaks; assignment of peaks was based on minimum difference of the means. Simple majority of individual peak assignments was used to determine the treatment type of unknown samples. The SVD process remained unchanged, except that the input matrices were generated by stacking different combinations of the Raman spectra. The first input matrix contained control Raman spectra (columns 1–50), EGF Raman spectra (columns 51–100) and unknown 1 (columns 101–110). The rows were the points of the spectral range, 500–2000 cm^−1^ (750 rows). The process was repeated for control, EGF and unknown 2; then control, EGF and unknown 3; control, FGF2 and unknown 1; control, FGF2 and unknown 2; control, FGF2 and unknown 3; EGF, FGF2 and unknown 1; EGF, FGF2 and unknown 2; and, finally, EGF, FGF2 and unknown 3.

## Results

### FGF2 promotes proacinar and ductal phenotype differentiation in salivary gland organoids

**Fig. 1:**
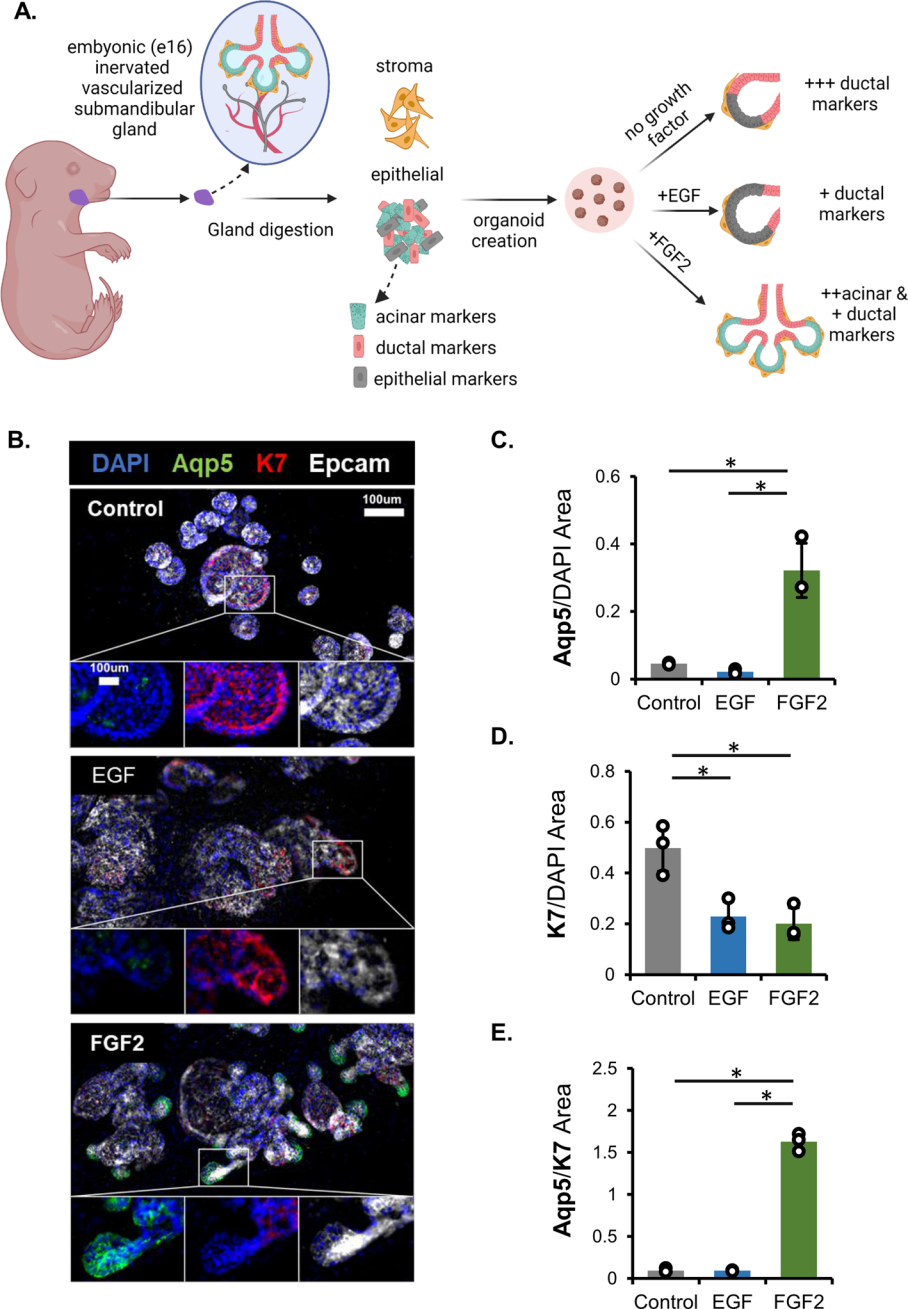
FGF2 promotes proacinar and inhibits ductal differentiation in embryonic salivary gland organoids. **A** Illustration of salivary gland organoid creation from murine embryonic salivary gland cells, which are separated for regulated recombination in Matrigel. Keratin-7 (K7)-positive ductal cells are shown in red, aquaporin-5 (AQP5)-positive pro-acinar cells are shown in green, and mesenchymal stromal cells are shown in yellow. Created with Biorender.com. **B** Representative images show that, while all organoids express the pan-epithelial marker EpCAM (white) and some level of the ductal marker K7 (red), the elaboration of AQP5^+^ cells (green) is restricted to branched organoids treated with FGF2. Nuclei are stained with DAPI. (**C**–**E**) Quantification of microscopy images with relative area ratios for AQP5 to DAPI, K7 to DAPI, and AQP5 to K7. Error bar represents 95% confidence interval, *n* = 3 technical replicates; asterisk indicates significance with *p* < 0.05 (one-way ANOVA with Tukey’s post hoc test) (statistical summary in Additional file 1: Table S1–S3)

Salivary gland organoids were formed from embryonic epithelial progenitor cells and mesenchymal stromal cells and were treated with medium containing fibroblast growth factor 2 (FGF2) to form proacinar organoids, epidermal growth factor (EGF), or control medium lacking growth factor (Fig. 1A), following our established protocols [2, 24, 25]. Organoids treated with FGF2, but not EGF-treated or those without growth factor supplementation, exhibited robust branching and proacinar cell differentiation, with buds expressing membrane-localized aquaporin-5 (AQP5) (Fig. 1B). Immunofluorescent antibody staining was performed to recognize epithelial cells expressing AQP5 to identify proacinar cells, and keratin-7 (K7) to identify ductal cells. As expected, the AQP5/DAPI ratio was significantly increased with FGF2 treatment (Fig. 1C). An enrichment of ductal cells (K7/DAPI ratio) was observed in the control, but not in the EGF- or FGF2-treated cells (Fig. 1D). The ratio of the image area of proacinar cells (AQP5) to ductal cells (K7) was significantly increased in FGF2-treated organoids (Fig. 1E). Neither untreated control nor EGF-treated organoids showed significant AQP5 levels, and both EGF- and FGF2-treated organoids have significantly less K7 compared with the untreated control. These data confirm the previously established organoid method [2, 24, 25], which requires both epithelial and stromal cells with FGF2 treatment in Matrigel-embedded organoids to produce AQP5-positive, proacinar differentiation in end buds and increased ductal cells in the absence of FGF2 or EGF.

### Development of Raman confocal method to evaluate salivary gland organoids

**Fig. 2:**
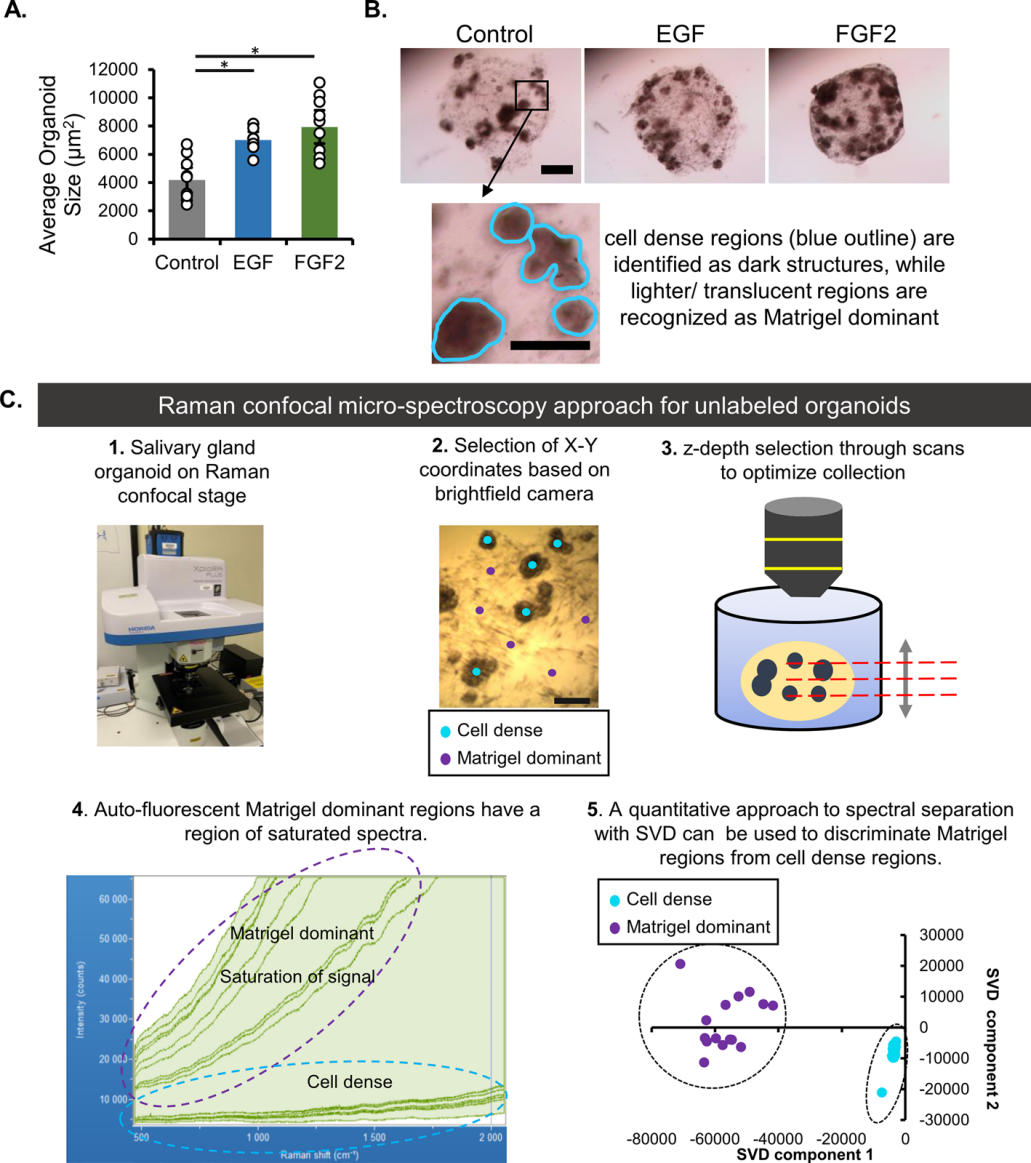
Development of Raman confocal microspectroscopy method to evaluate salivary gland organoids. **A** Quantification of organoid size based on area. *n* = 7 technical replicates across three experiments (statistical summary in Additional file 1: Table S4). **B** Representative images of control and growth factor-treated organoids prior to Raman imaging, with the cell-dense, largely epithelial regions highlighted in zoomed panel. Scale bar, 500 µm (panel), 250 µm (zoomed region). **C** Visualization of the Raman confocal microspectroscopy method for identification of cell-dense regions. This method allows the separation of cell dense-organoid regions from Matrigel-dominant, or stromal, regions, which can be done either manually by peak comparison or with a multivariate SVD approach

Raman imaging of organoids is complicated by a pervasive Raman signal from culture surfaces and ECM or other scaffolding materials. Here, a method employing confocal Raman microspectroscopy was developed to capture only Raman photons originating from the cell-dense, primarily epithelial, regions of the organoids and not from cells or proteins in the surrounding matrix. These studies were first performed in hydrated, intact, fixed organoids to determine the appropriate amount of sampling required to classify the organoids. To eliminate signals from plastic culture dishes, the unlabeled organoids were transferred to a quartz-based chamber, and the organoids remained submerged in 1× PBS for imaging. The average area of the fixed organoid was determined while in the culture dish, and it was shown to be larger for the growth factor-treated organoids compared with untreated organoids (Fig. 2A). Upon transfer to the quartz-based chamber, the cell-dense regions, representing regions composed primarily of epithelial cells, were identifiable and distinct from the Matrigel-dense regions, representing stromal cell-rich regions (Fig. 2B). After the *X*–*Y* coordinates of the cell-dense regions were identified using brightfield imaging, a series of Raman confocal measurements along the *Z*-direction was performed to focus on the cell-dense volume and exclude the spectra from the highly fluorescent Matrigel. The laser and acquisition settings were selected to facilitate the easy separation of Matrigel-rich regions from cell-dense regions (Fig. 2C).

### FGF2-treated organoids have a Raman signature that is distinct from untreated or EGF-treated organoids

**Fig. 3:**
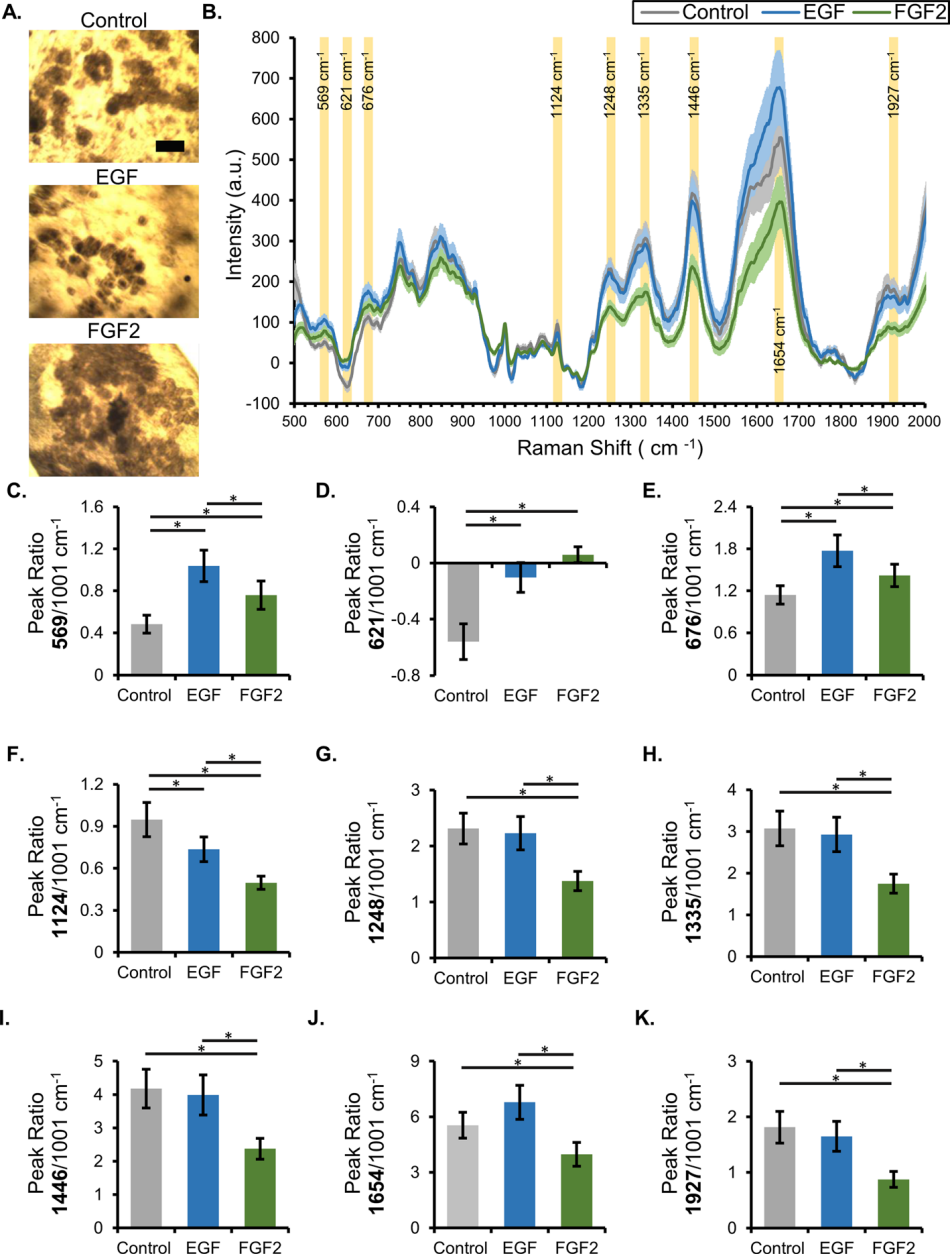
FGF2-treated organoids have a Raman signature that is distinct from EGF-treated or untreated organoids. **A** Representative brightfield images of fixed organoids on the Raman confocal stage; the cell-dense regions (dark brown) are easily distinguished from the Matrigel-dominant area (scale bar, 200 µm). **B** Average Raman spectra for cell-dense regions of control and EGF- and FGF2-treated organoids, with error bars representing the 95% confidence interval. **C**–**K** Quantification of select peak intensities in the spectral dataset based on the ability to differentiate one or more treatments (*n* = 50 separate spectra collected from a minimum of three independent organoids; representative experiment * asterisk indicates significance with *p* < 0.05; statistical summary in Additional file 1: Table S5)

Differentiation state-specific spectra for each organoid were determined following the rejection of the Matrigel spectra. Organoids were treated with growth factors or control medium for 7 days, fixed with 4% PFA, and transferred in an intact, hydrated form to quartz slides for imaging. Raman measurements were collected from the cell-dense, epithelial-enriched regions of the organoids, which appear as dark-brown spheres in brightfield images (Fig. 3A), and were subjected to postprocessing and removal of the Matrigel spectra. The average Raman spectra for control (untreated), EGF-treated, and FGF2-treated organoids were distinct from each other, with several peaks having significant intensity differences (Fig. 3B). Select peak ratios could be used to differentiate at least one organoid treatment from the other two (highlighted in Fig. 3B, analysis in Fig. 3C–K). Some peak ratios showed significant differences between all groups (Fig. 3C, E, F), while others could only separate one treatment from the other two (Fig. 3D, G–K). Many of these peaks have previously been assigned to specific compounds and cellular components, with known specific peak assignments indicated in Table 1, and broad peaks in Additional file 1: Fig. S1. Specifically, there are large changes in amide III, CH_3_-CH_2_ wagging, CH_2_ deformation, and amide II region of the Raman spectra (Additional file 1: Fig. S1). These regions are highly associated with alterations in proteins and lipids of biological samples [7]. These data demonstrate that there are specific ratios in Raman peaks that vary between organoids in different differentiation states.

**Table 1:** Raman peak assignments reference table

Peak (cm^−1^)	Peak assignment (specific wavenumber reference in parentheses)	References
569	Tryptophan/cytosine, guanine (573 cm^−1^) inositol residue in phosphatidylinositol (576 cm^−1^)	[38, 39]
621	C–C twisting mode of phenylalanine (621 cm^−1^), tyrosine	[38]
676	Ring breathing modes in the DNA bases (674 cm^−1^) Guanine ring breathing (679 cm^−1^)	[38, 40]
745	DNA/tryptophan	[7]
920	C–C stretch of proline ring/glucose Collagen	[7, 15]
1003	Phenylalanine (1001–1004 cm^−1^)	[40]
1124	C–C stretching mode of lipids/protein C–N stretch/glucose	[38]
1248	Amide III (collagen) (CH2 wag, C–N stretch)/pyrimidine bases (C, T)	[38]
1335	CH3CH2 twisting mode of protein, collagen (1335–1345 cm^−1^) CH3CH2 deformation/wagging nucleic acid (1335–1339 cm^−1^)	[7, 38]
1446	Phospholipid bands (1437–1442 cm^−1^)	[39]
1654	Lipid (C=C stretch) (1652–1655 cm^−1^) Amide I (1654–1659 cm^−1^)	[38, 40, 41]
1926	CO stretching band	[42]

Select Raman peaks associated with quantification, tentative peak assignments, and references

**Fig. 4:**
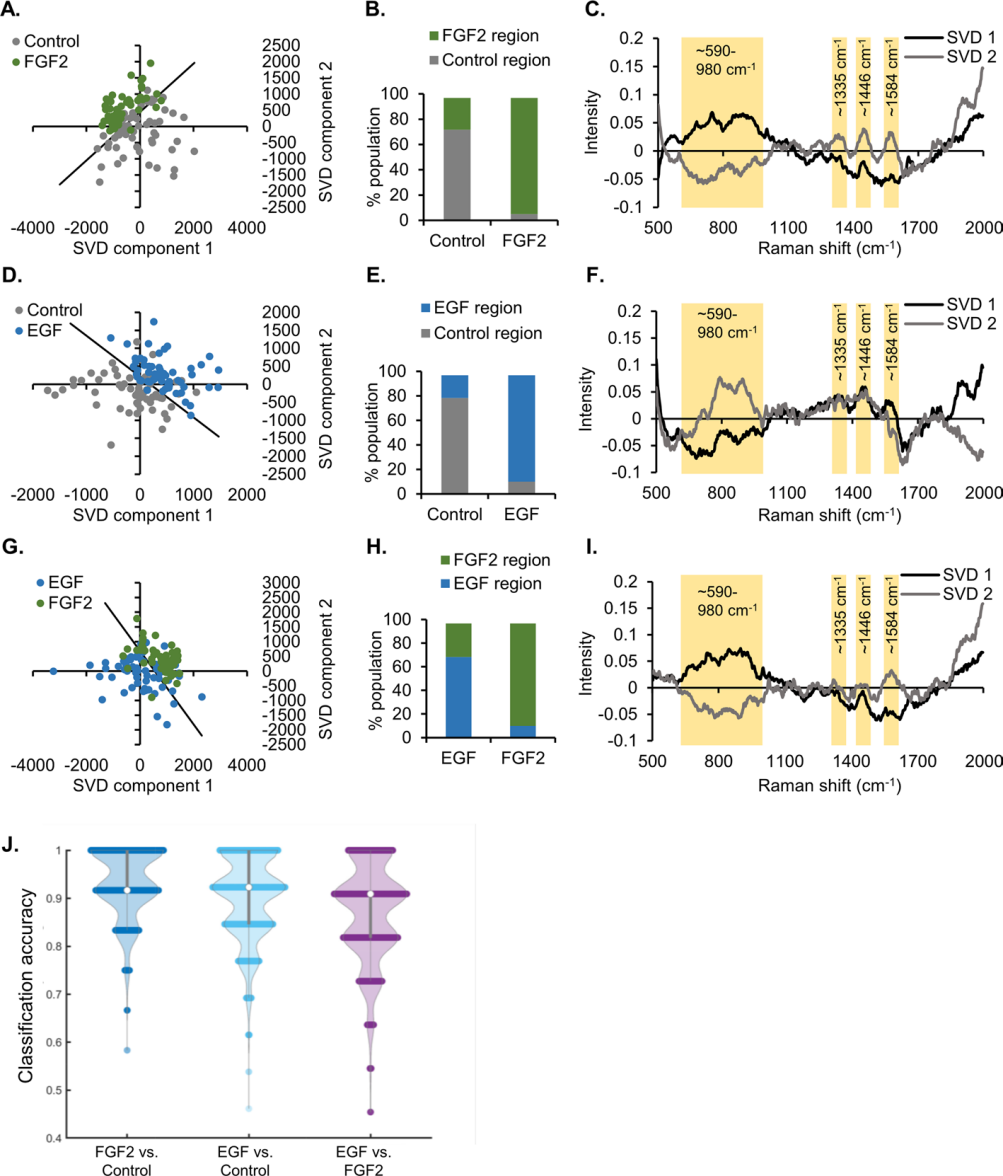
Multivariate and machine learning analysis of Raman spectra from fixed organoids facilitates separation of untreated and EGF- and FGF2-treated organoids. **A–I** Multivariate SVD analysis of data from Fig. 3 with control versus FGF2 (**A**–**C**), control versus EGF (**D**–**F**), and EGF versus FGF2 (**G**–**I**). For each comparison, there is an SVD plot where each dot represents a single spectrum, with clustering of similar spectra (**A, D, G**). A line in each SVD plot is placed perpendicular and equidistant to the mean of each treatment to separate the treatments in an unbiased manner. The distribution of the spectra in each region is plotted in **B**, **E**, and **H**, while the SVD components producing the distribution are plotted in **C**, **F**, and **I**. Select regions of interest within the component graphs are highlighted in yellow (*n* = 50 separate spectra collected from a minimum of three independent organoids, representative experiment). **J** Classification accuracy of machine learning (RSVM) on fixed Raman organoid mixed datasets, as indicated, and displayed as a violin plot (120 spectra per comparison; standard deviation less than 0.10 for all comparisons)

In addition to the analysis of individual peak ratios, Raman spectral data were analyzed by both multivariate analysis and supervised machine learning algorithms. The entire spectral dataset was subjected to SVD analysis (Fig. 4A–I). The SVD analysis facilitates the unbiased processing of large spectral datasets. The spectra with similar features cluster together on the SVD scatter plot, while the SVD components provide insight into the specific spectral regions that differentiate the samples [19]. A line systematically placed in each SVD scatter plot (Fig. 4A, D and G) that is perpendicular and equidistant to the individual mean of each cluster quantifies the separation between treatments (Fig. 4B, E and H). The qualitative analysis shows that, within each comparison made, there is clustering together based on spectra from the same treatment group (Fig. 4A, D, G). Quantification of the data shows that FGF2 and EGF treatments have 10–20% overlap compared with control (Fig. 4B, E). The comparison of the two growth factor treatments results in 10–30% of spectra being inseparable (Fig. 4H). This is not unexpected, since all samples started with the same cellular material and some ductal cells are present in organoids with all treatments, while proacinar differentiation is observed only in the FGF2-treated samples. The leading SVD components share similar trends, as expected, because the broad peaks represent structures that are highly abundant in biological samples (Fig. 4C, F and I). The highlighted regions have notable variations between the treatment groups. Not all peaks that are prominent in the SVD components correspond to the peaks selected for analysis of peak ratios (Fig. 4G–K). This shows that the multivariate analysis is more sensitive and contains additional information that is not limited to the several well-defined peaks used for the peak ratio analysis. For example, the very broad peaks spanning 100–200 wavenumbers have significant variations between treatments.

Machine learning classifiers trained from kernel-based support vector machines are capable of classifying samples from small, high-dimensional spectral datasets [31–33]. More specifically, the more recently developed robust support vector machines (RSVM) were selected to classify the Raman datasets as their learned classifiers are more resistant to outliers [29]. The fixed organoid Raman spectral data were subjected to RSVM analysis. Each comparison group consisted of 120 spectra, with 100 used for training and 20 reserved for classification. These spectra data are normalized as follows: at each wavenumber, we subtracted the minimum of the intensity values across different spectra from each intensity value, and then we divided the difference by using the range of the intensity values at the same wavenumber. As a result of this normalization step, all the intensity values lie within the range of 0–1. RSVM was then implemented on these normalized spectra data, and this process was repeated 4000 times. The classification accuracy of the fixed organoid comparison is displayed as a violin plot (Fig. 4J). The accuracy was 94% for FGF2 versus control, 87% for EGF versus control, and 90% for EGF versus FGF2. This indicates RSVM’s potential utility in routine Raman spectral classification approaches.

### Blinded organoid samples successfully identified with two distinct approaches

**Fig. 5:**
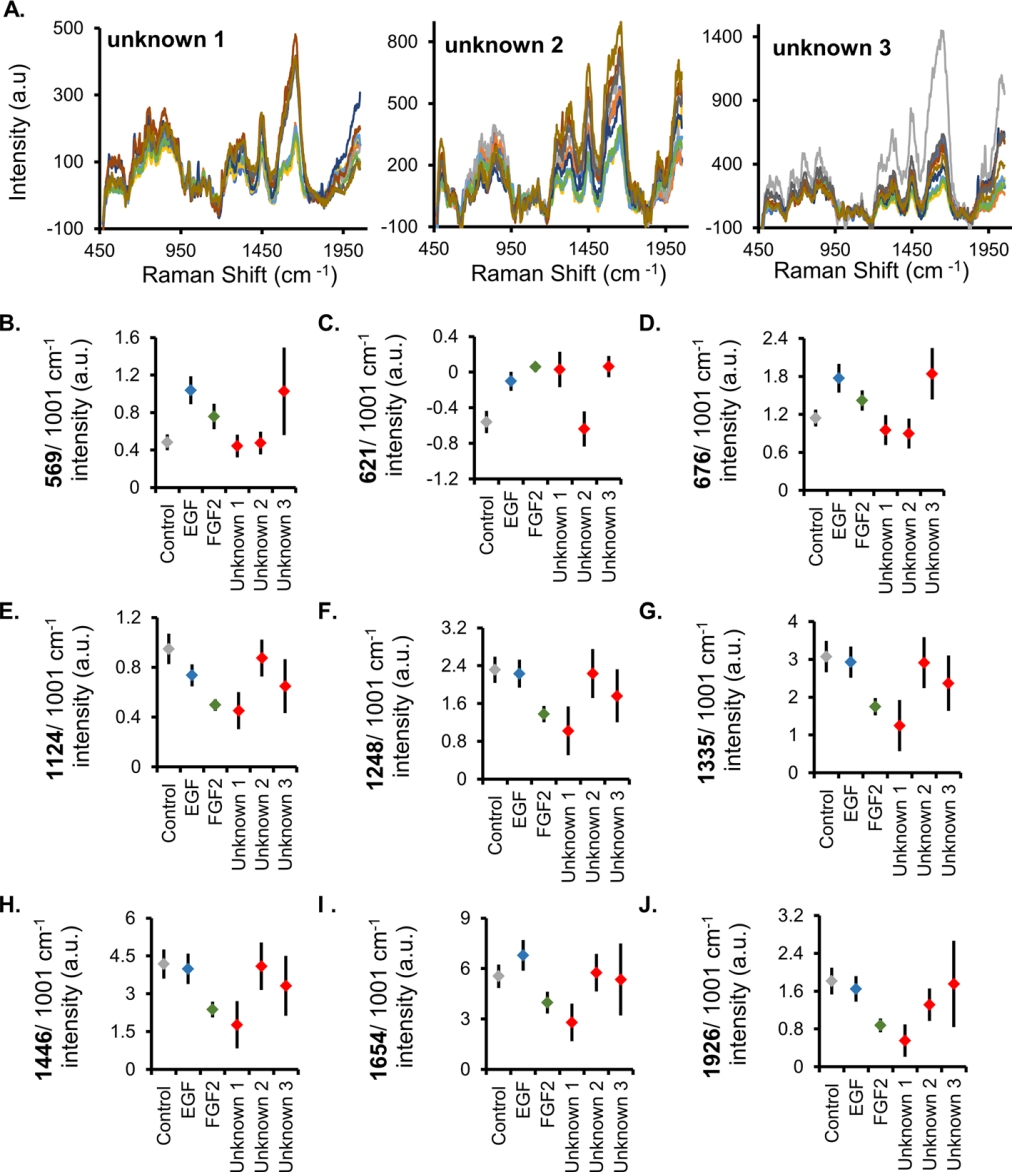
Unknown organoids identified using analysis of select Raman spectral peaks. **A** Raman spectra of cell-dense organoid region for each unknown (blinded) organoid dataset (ten processed, normalized spectra per treatment). **B**–**J** Comparison of the mean Raman peaks between the known samples (full spectra in Fig. 3) and the unknown datasets. Error bars equal 95% confidence interval. Table 2 details the assignment of the unknown peaks to specific treatments

To determine whether organoid differentiation state can be predicted using only the Raman signature, a blinded study was performed in which ten distinct spectra obtained from three unknown samples were assigned a control, EGF-treated, or FGF2-treated phenotype (Fig. 5A). Two separate approaches were utilized, one based on the multiple-peak comparisons and the other based on the SVD analysis. Multiple-peak comparisons used the mean ratios of specific Raman peaks, which included 569, 621, 676, 1124, 1248, 1335, 1446, 1654, and 1926 cm^−1^, all previously identified as showing significant differences between organoid phenotypes (Fig. 3B–K). The unknown sample spectra were averaged and normalized, and their mean ratios were compared with the known Raman peak ratios (Fig. 5B–J). Following the assignment of individual peaks based on the mean peak ratios, a simple majority was used to classify the unknown samples (Table 2). All unknown organoid samples were correctly identified as control, EGF-treated, or FGF2-treated using multiple Raman peak comparisons.

**Table 2:** Determination of unknown samples using two distinct approaches

(A) Unknown organoid assignments by evaluating multiple peaks: nine peaks individually evaluated for each unknown organoid dataset, the peak assignment determined by mean proximity, with inclusion of significantly overlapping ranges noted in parentheses (see individual peak graphs in Fig. 4). Final unknown assignment determined by simple majority			
Peak wavenumber (cm^−1^)	Unknown 1	Unknown 2	Unknown 3
569	Control	Control	EGF (FGF2)
621	FGF2 (EGF)	Control	FGF2
676	Control	Control	EGF (FGF2)
1124	FGF2	Control	EGF (FGF2)
1248	FGF2	EGF (control)	FGF2 (EGF)
1335	FGF2	Control (EGF)	EGF
1446	FGF2	Control (EGF)	EGF
1654	FGF2	Control	Control
1926	FGF2	EGF	Control (EGF)
Final assignment based on multiple peak analysis→	Unknown 1 = FGF2 (seven out of nine individual peaks assigned as FGF2)	Unknown 2 = control (seven out of nine individual peaks assigned as control)	Unknown 3 = EGF (five out of nine individual peaks assigned as EGF)
(B) Unknown organoid assignments by SVD analysis: spectra of unknown organoid dataset subjected to SVD analysis with known samples. Line formed between clusters runs perpendicular to the mean of each cluster (Fig. 5). Assignment of unknown based on percent of unknown spectra distribution with known samples (majority of population determines assignment, Fig. 5)			
Final assignment based on SVD analysis→	Unknown 1 = FGF2 →100% of unknown 1-spectrum cluster in FGF2 region compared with EGF (Additional file 1: Fig. S2 shows all comparisons)	Unknown 2 = control →90% of unknown 2-spectrum cluster in control region compared with EGF (Additional file 1: Fig. S3 shows all comparisons)	Unknown 3 = EGF →100% of unknown 3-spectrum cluster in EGF region compared with control (Additional file 1: Fig. S4 shows all comparisons)
(C) Actual assignment of unknown organoid datasets (unblinding of samples)			
	Unknown 1 = FGF2	Unknown 2 = control	Unknown 3 = EGF

**Fig. 6:**
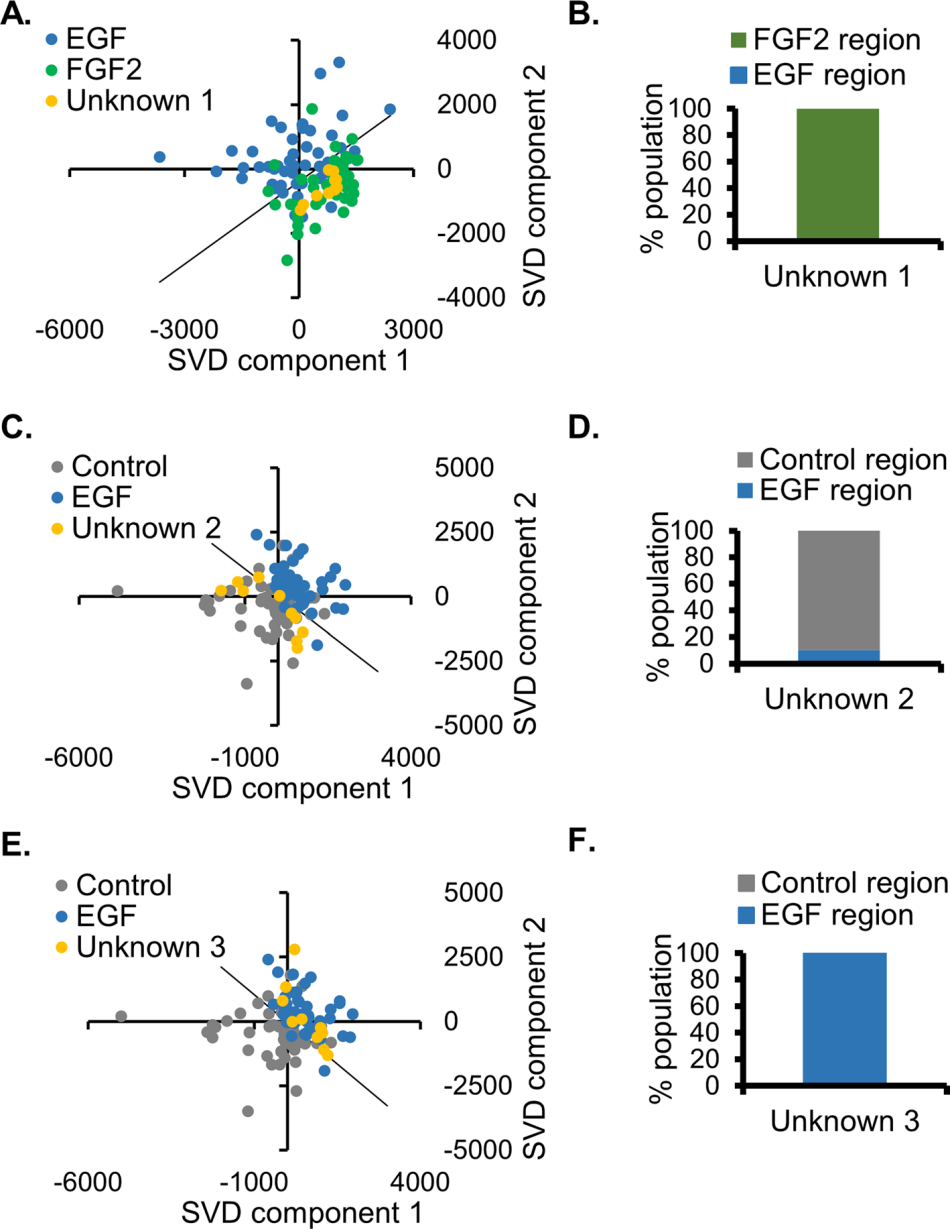
Unknown organoids identified using SVD analysis of Raman spectra. Raman spectra derived from the unknown (blinded) datasets of cell-dense organoid regions were subjected to SVD analysis with known FGF2 (green), EGF (blue), and control (gray) datasets (derived from Fig. 3). One representative plot is shown for each unknown, which demonstrates codistribution with a known treatment. **A** SVD plot of unknown 1 with known EGF and FGF2 spectra. **B** Percentage of population of unknown 1 codistributed with FGF2 or EGF. **C** SVD plot of unknown 2 with known EGF and control spectra. **D** Percentage of population of unknown 2 codistributed with EGF or control. **E** SVD plot of unknown 3 with known EGF and control spectra. **F** Percentage of population of unknown 2 codistributed with EGF or control (specific assignments of unknown samples detailed in Table 2). Additional SVD comparisons for unknowns 1–3 are provided in Additional file 1: Fig. S2–S4, respectively

An alternative approach for identifying unknown organoid samples utilized the multivariate SVD analysis that was also used in the evaluation of Raman signatures of organoid phenotypes (Fig. 4A–I). Each unknown sample, consisting of ten spectra, was compared individually with the known datasets (Fig. 6). A significant overlap was seen in the SVD plots (Fig. 6A, C, E and Additional file 1: Figs. S2–S4) and the ratios of spectra codistributed with each known phenotype were computed (Fig. 6B, D, F). The final assignment of each unknown organoid is included in Table 2. The correct assignment of unknown organoids was made with both peak-intensity analysis and SVD approaches. All unknown organoid samples were correctly identified as control, EGF-treated, or FGF2-treated using multivariate SVD analysis of their Raman signals.

### Live organoids have distinct Raman signatures

**Fig. 7:**
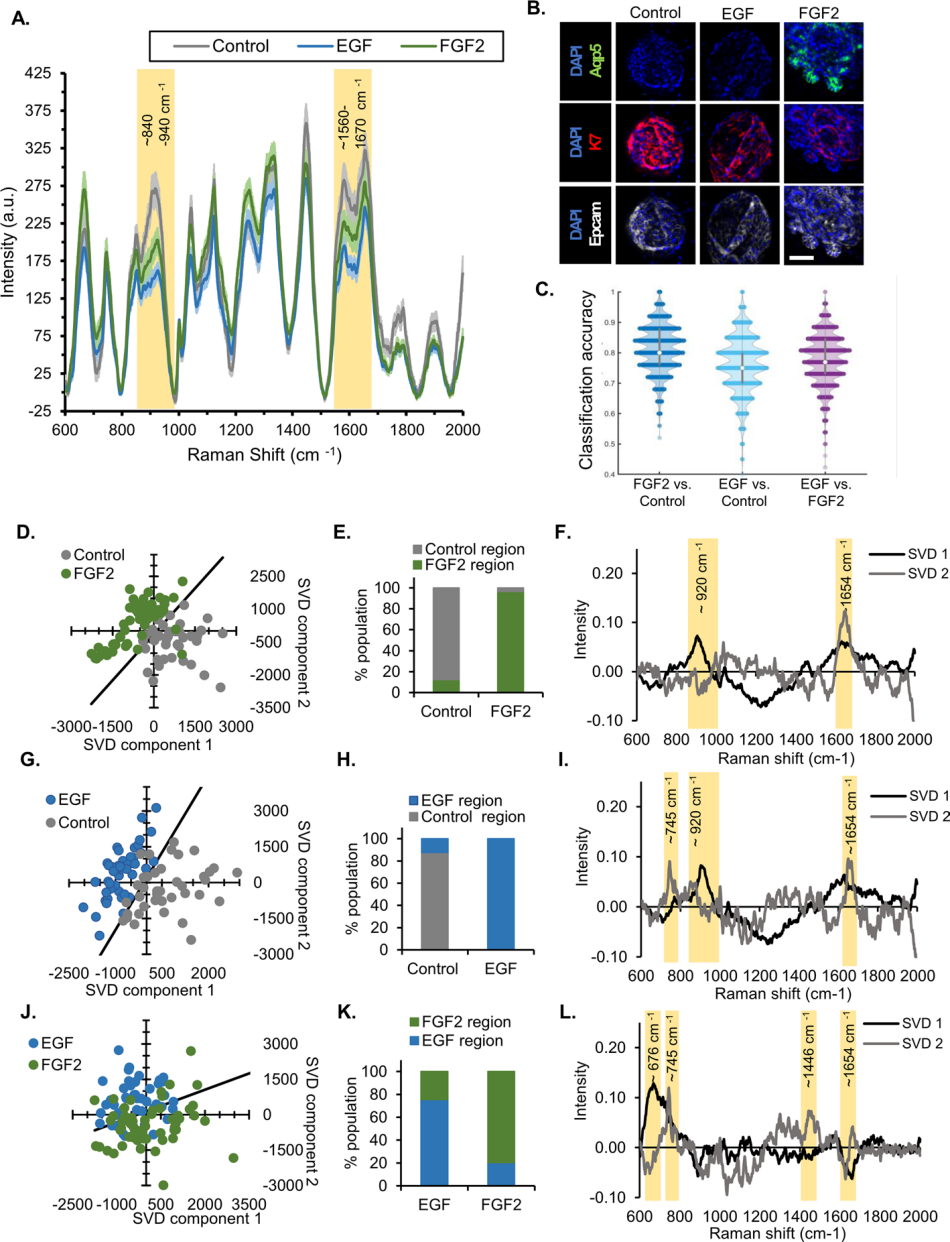
FGF2-differentiated live organoids with distinct Raman signature. **A** Average Raman spectra for cell dense regions of control, EGF-treated, and FGF2-treated organoids, with error bars representing the 95% confidence interval. **B** Representative images of salivary gland organoids that were first subjected to live Raman imaging prior to fixation and immunostaining to confirm the proacinar (AQP5) cells in FGF2-treated organoids, additional stains for epithelial cells (EpCAM), ductal cells (K7), and nuclei (DAPI). Scale bar, 100 µm. Larger images and quantification shown in Additional file 1: Fig. S5. **C** Classification accuracy of machine learning (RSVM) on live Raman organoid dataset (228–267 spectra per comparison, Standard deviation less than 0.10 for all comparisons). **D**–**L** Multivariate SVD analysis of representative experiment with control versus FGF2 (**D**–**F**), control versus EGF (**G**–**I**), and EGF versus FGF2 (**J**–**L**). For each comparison, there is an SVD plot where each dot represents a single spectrum, with clustering of similar spectra (**D**, **G**, **J**). The distribution of the spectra in each region is plotted in **E**, **H**, and **K**, while the SVD components producing the distribution are plotted in **F**, **I**, and **L**. Select regions of interest within the component graphs are highlighted in yellow (*n* = 40+ separate spectra collected from a minimum of three independent organoids per condition, representative experiment shown of three replicate experiments)

Assessing the ability of Raman spectroscopy to be used for classification of live organoids was important as it could be utilized to confirm the differentiation state of samples prior to experimental manipulations. Live organoids were prepared and cultured in the presence of either EGF or FGF2, or in the absence of exogenous growth factor. Organoids were kept undisturbed in their glass-bottomed culture dishes in phenol red-free medium for Raman imaging. Live organoid Raman imaging was similar to that of fixed organoids, although imaging time was reduced and the confocal pinhole diameter was increased, as detailed in methods. In addition, the sampling location was limited to the upper *Z*-depths of organoids, which allowed the organoids to remain in the glass-bottom culture dishes and within medium during imaging. Live untreated, EGF-treated, or FGF2-treated organoids produced Raman spectra with notable variations between conditions in the C–C stretching (840–940 cm^−1^) and amide 1 (1560–1670 cm^−1^) Raman shift regions (Fig. 7A) that were similar, but not identical, to the fixed organoids, as expected ^7^. Other groups also describe that diagnostically significant Raman spectral regions differ between formalin-fixed tissue and live or frozen tissues [7, 34].

Compared with fixed organoids, the unprocessed spectra of live organoids showed differences in background and noise, and both were addressed with modification of basic postprocessing, as detailed in the methods. Increased noise was not unexpected since the imaging time had been reduced, and the samples were live and undergoing respiration and growth. After Raman imaging, the organoids were fixed and subjected to immunostaining to confirm the proacinar differentiation of FGF2-treated organoids with detection of AQP5 (Fig. 7B, Additional file 1: Fig. S5). As with the fixed organoids, the live dataset was processed with RSVM. The RSVM’s classification accuracy for FGF2 versus control was 81%, EGF versus control was 76%, and EGF versus FGF2 was 78% (Fig. 7C). Additionally, live organoid Raman spectra were subjected to SVD analysis, which showed that control (untreated), EGF-treated, and FGF2-treated organoids could be separated in a quantitative manner (Fig. 7D–L). Notable SVD component regions were highlighted in Fig. 7F, I, and L, and the associated peak assignments are listed in Table 2. These data demonstrate that live organoids can be imaged noninvasively with Raman spectroscopy, and that live FGF2-induced proacinar salivary gland organoids have a unique Raman signature that differs significantly from untreated or EGF-treated organoids.

## Discussion

Organoid models are popular because they simulate the 3D cellular organization within an organ and the signaling between cells for purposes of understanding organ development and for many translational applications [1, 2]. However, the inherent heterogeneity in their development demands nondestructive methods to verify the differentiation state or phenotype of the organoids. Here, we report a Raman-based imaging method that can be used to distinguish between organoids in different states that are imaged either fixed or live. We find that Raman microspectroscopy is ideal for organoid imaging, since the confocal microscope can focus exclusively on cell-dense regions, reject out-of-focus light, and allow postprocessing removal of saturated exogenous ECM-rich spectra. Unique Raman spectral signatures, which represent the average of multiple cellular regions, can then be defined for the desired developmental stage or differentiated phenotype in the context of live 3D organoids.

In this study, we focused on the cell-dense regions of organoids enriched for epithelial cells, and the Raman signatures associated with different epithelial differentiation states. Importantly, Raman microspectroscopy facilitated the correct identification of organoid treatments using two distinct methods handling blinded datasets. Interestingly, the differentiation and organization of the proacinar cells within the FGF2-fixed samples produced the changes in the average spectra at broad peaks associated with amide III, CH deformation, and amide I. These data may be indicative of a differential distribution and organization of lipids and proteins in cells undergoing secretory proacinar differentiation. These data also indicate that a higher throughput in identification and/or classification of either the live or fixed organoids is achievable by combining Raman imaging with machine learning (RSVM) and multivariate data analysis, such as SVD.

In general, and especially for our live organoid studies, SVD was an excellent method for identifying the important features that can differentiate the organoids under different treatments, even when the noise increased with live-organoid imaging. Machine learning is another useful tool for categorizing the spectra when there are a sufficient number of spectra. Machine learning may save time in processing over SVD when using high-throughput assays; however, the accuracy may be lower with small datasets, as we observed in this study. The utilization of Raman spectroscopy to differentiate between healthy and diseased tissue in numerous animal and human models has been well established, with far-reaching clinical applications [7, 12, 13, 35–37].

## Conclusion

We report a strategy to classify organoid differentiation state with Raman confocal microspectroscopy. This unique approach is focused on capturing the Raman spectral signatures from cell-dense epithelial cell regions of organoids. Although organoids are essential tools utilized across many biological and bioengineering disciplines, label-free nondestructive methods needed to assess organoid responses have been lacking. The use of Raman spectroscopy for quality control is far more robust than a select set of markers, and it does not require additional chemicals, making it an appropriate technique for future application with clinical samples.

We demonstrated the robustness of this approach using classification approaches, including machine learning and SVD. Our data show that we can identify unknown sample spectra in blind studies. Although we developed the methods with formalin-fixed organoids, we also show that the approach is effective in live organoids. As the live organoids were unlabeled, intact, and imaged in submerged medium in their original culture dish, the approach can be easily adapted to high-throughput monitoring applications.

Our approach is novel as it focuses on unlabeled, nondestructive imaging of intact organoids, includes an original method of rejecting the Matrigel-dense signals, and defines Raman signatures for differentiated organoids in either fixed or live states. It can be converted into a high-throughput screening method to ensure conformity in organoid development without sample destruction and determine thresholds for production of more uniform samples. The comprehensive and holistic Raman spectra approach provides significantly more information than a small set of quality-control (QC) markers. This approach is promising for numerous translational and preclinical organoid models that cannot utilize fluorescent or other labels to probe their differentiation state or response to manipulation, and thus provides a more reliable QC tool for assessing organoid differentiation state even though there is some uncertainty regarding what the spectra represent biologically. As material and labor costs of routine organoid production rise, Raman screening of organoids could become a cost-effective quality-control tool.

## Supplementary Information

**Additional file 1: Figure S1.** Raman Spectra with common broad peak assignments. **Figure S2.** Unknown 1- SVD comparisons with known datasets. **Figure S3.** Unknown 2- SVD comparisons with known datasets. **Figure S4.** Unknown 3- SVD comparisons with known datasets. **Table S1.** Statistical summary for Fig. 1C. **Table S2.** Statistical summary for Fig. 1D. **Table S3.** Statistical summary for Fig. 1E. **Table S4.** Statistical summary for Fig. 2A. **Table S5.** Statistical summary for Fig. 3C–K.

## Data Availability

The datasets used and/or analyzed during the current study are available from the corresponding author on reasonable request.

## Abbreviations

AQP5: Aquaporin 5, a proacinar cell marker
DAPI: 4′,6-Diamidino-2-phenylindole, a nucleic acid dye
DMEM/F12: Dulbecco’s Modified Eagle Medium/Nutrient Mixture F12
EGF: Epidermal growth factor
EpCAM: Epithelial cell adhesion molecule, epithelial cell marker
FBS: Fetal bovine serum
FGF2: Fibroblast growth factor 2
FITC: Fluorescein isothiocyanate
K7: Keratin 7, a ductal cell marker
Pen-Strep: Penicillin and streptomycin
