# Supplementary material for: Raman microspectroscopy fingerprinting of organoid differentiation state
Source: Cell Mol Biol Lett. 2022 Jun 28;27:53. doi: 10.1186/s11658-022-00347-3 (PMC9238268; doi:10.1186/s11658-022-00347-3)
Supplement: Supplementary file 1 — Additional file 1: Figure S1. Raman Spectra with common broad peak assignments. Figure S2. Unknown 1- SVD comparisons with known datasets. Figure S3. Unknown 2- SVD comparisons with known datasets. Figure S4. Unknown 3- SVD comparisons with known datasets. Table S1. Statistical summary for Fig. 1C. Table S2. Statistical summary for Fig. 1D. Table S3. Statistical summary for Fig. 1E. Table S4. Statistical summary for Fig. 2A. Table S5. Statistical summary for Fig. 3C–K. [file 11658_2022_347_MOESM1_ESM.docx]

Supplementary Materials for

**Raman Microspectroscopy Fingerprinting of Organoid Differentiation State**

**Authors**

Kate Tubbesing^1^, Nicholas Moskwa^2,3^, Ting Chean Khoo^1^, Deirdre A. Nelson^2,3^, Anna Sharikova^1^, Melinda Larsen^2,3,^ *, Alexander Khmaladze^1,^*

*Corresponding author. Email: mlarsen@albany.edu and akhmaladze@albany.edu

**This PDF file includes:**

Supplementary Figures

Supplementary Figure 1. Raman Spectra with common broad peak assignments.

Supplementary Figure 2. Unknown 1- SVD comparisons with known datasets.

Supplementary Figure 3. Unknown 2- SVD comparisons with known datasets.

Supplementary Figure 4. Unknown 3- SVD comparisons with known datasets.

Supplementary Tables

Supplementary Table 1. Statistical summary for Figure 1C

Supplementary Table 2. Statistical summary for Figure 1D

Supplementary Table 3. Statistical summary for Figure 1E

Supplementary Table 4. Statistical summary for Figure 2A

Supplementary Table 5. Statistical summary for Figure 3C-K


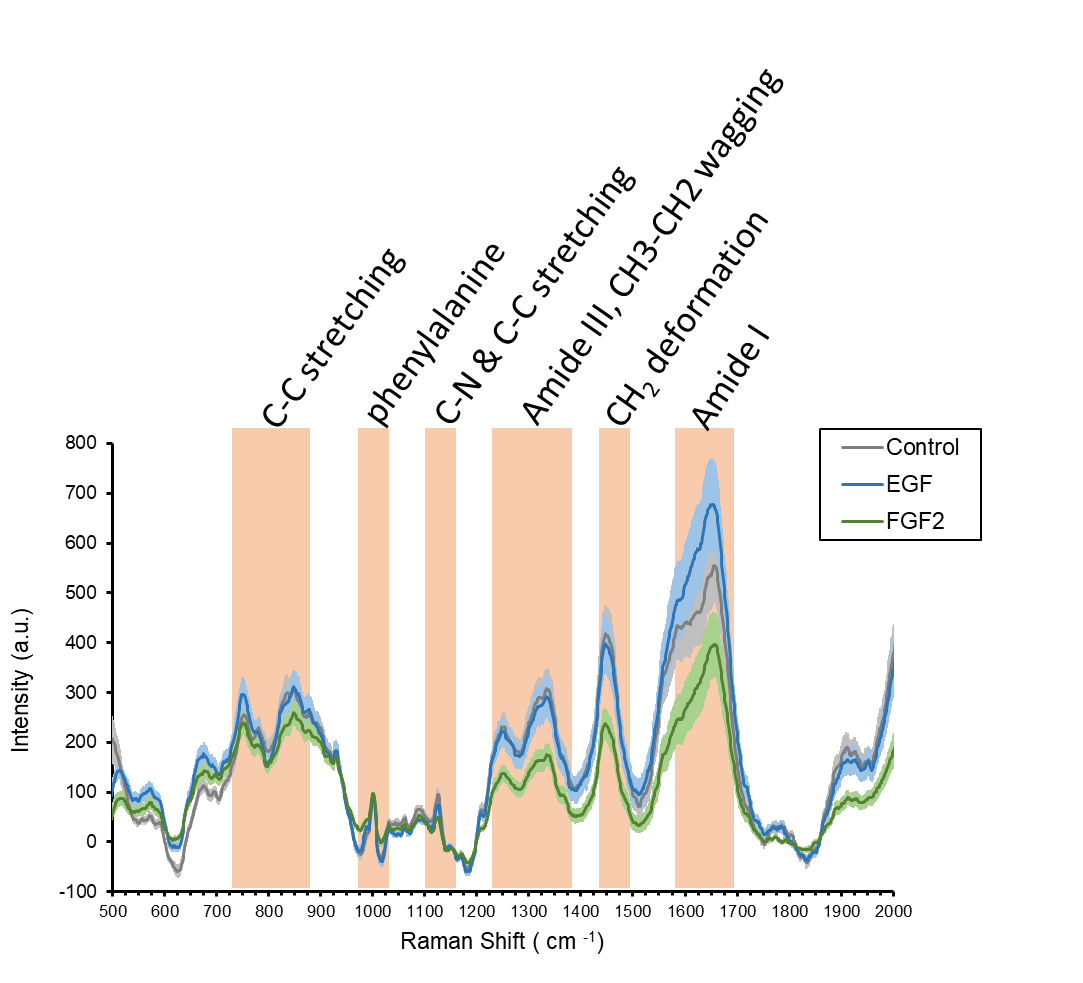
**Supplementary Figure 1.** Raman Spectral graph from Figure 3B with common peak assignments based on molecular composition

.

**Supplementary Figure 2.** Unknown 1- SVD comparisons with known datasets. For each comparison made there is the SVD scatter plot (A, C, D) and corresponding distribution of the unknown spectra based on scatter plot ( B, D, F).

*A-B are duplicated from Figure 6A and 6B.*


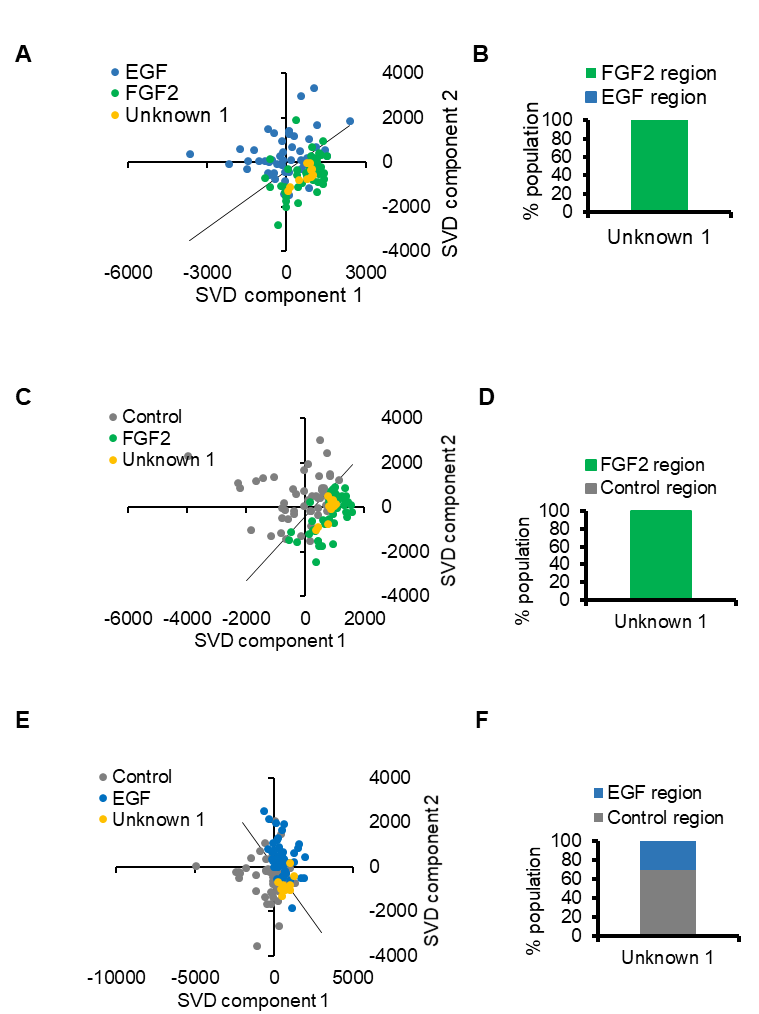


**Supplementary Figure 3.** Unknow 2 -SVD comparisons with known datasets. For each comparison made there is the SVD scatter plot (A, C, D) and corresponding distribution of the unknown spectra based on scatter plot ( B, D, F).

*A-B are duplicated from Figure 6C and 6D.*


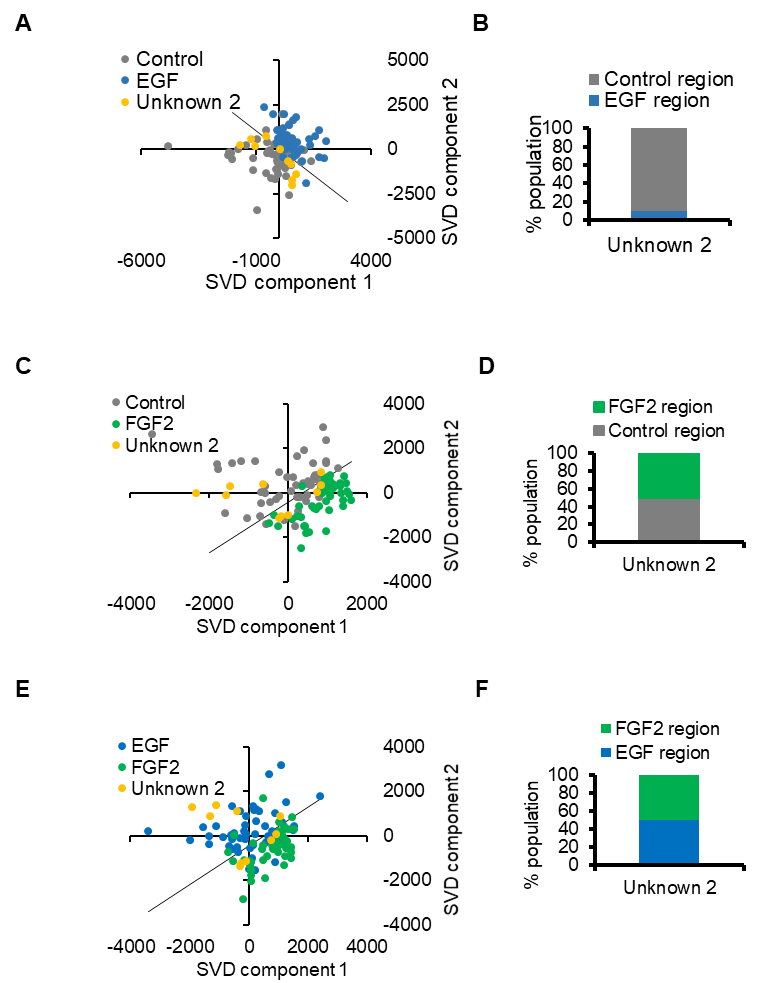


**Supplementary Figure 4.** Unknow 3 -SVD comparisons with known datasets. For each comparison made there is the SVD scatter plot (A, C, D) and corresponding distribution of the unknown spectra based on scatter plot ( B, D, F).

*A-B are duplicated from Figure 6E and 6F*


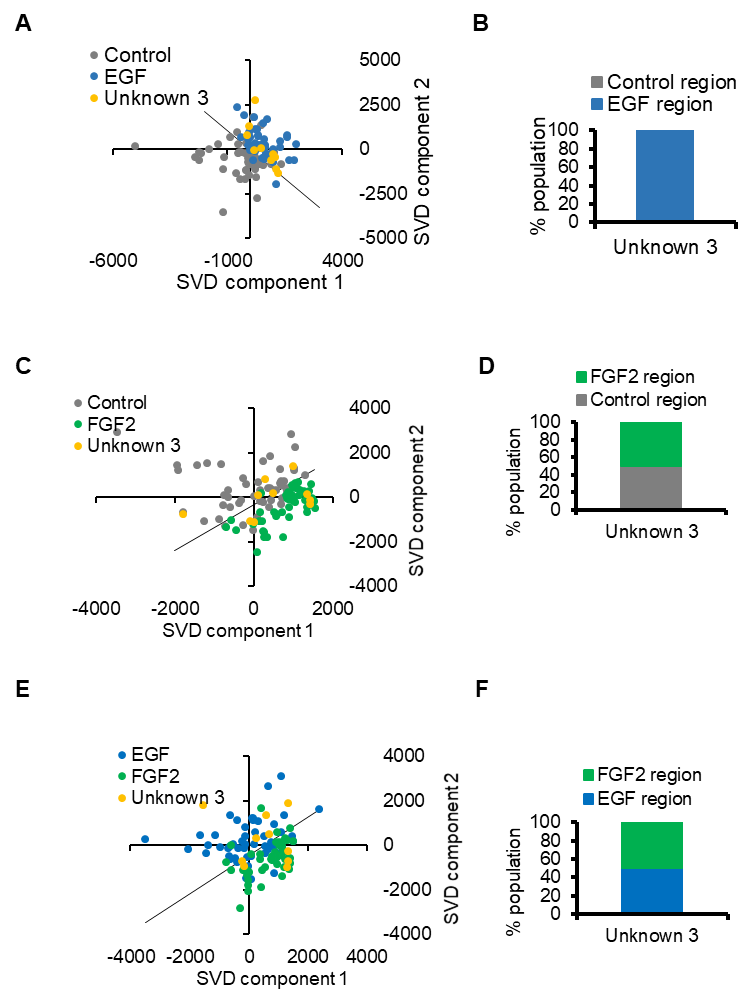


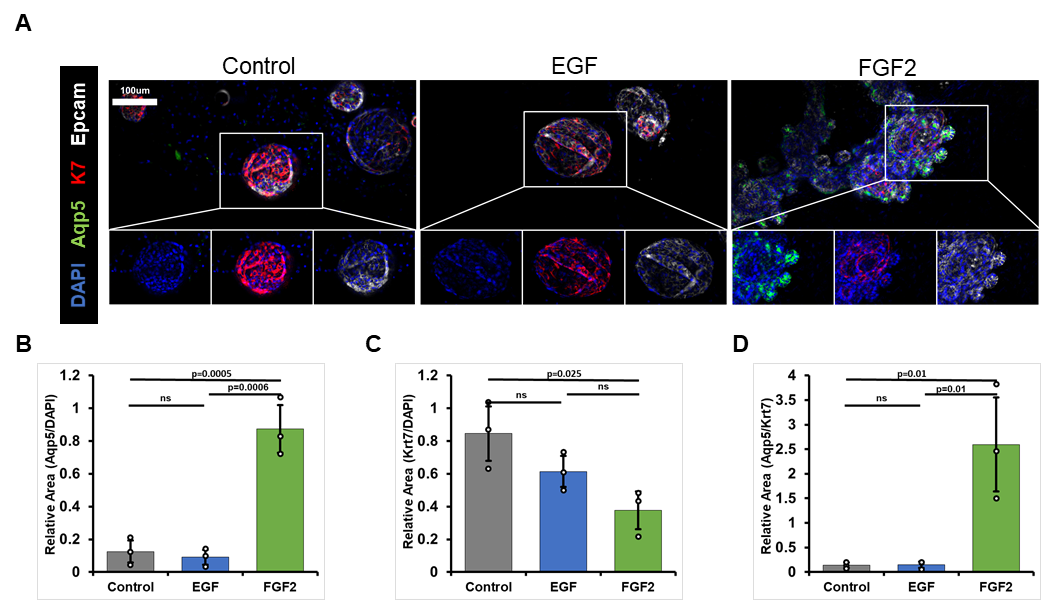
**Supplementary Figure 4.**  The zoomed-out images of figure 7B and associated quantification of organoid dataset which was imaged lived prior to fixation. **A**) Immunofluorescent images of zoomed out images from 6B. **B-D)** Quantification of immunofluorescent staining with the relative ratios of **B)** Aqp5/DAPI **C)** K7/DAPI and **D)** Aqp5/K7. N= 3 technical replicates with statistical analysis with Anova and a Tukey post-hoc.

**Supplementary Table 1.**

| **Statistical summary for Figure 1C.** Asterisk indicates statistical significance. | | | | | | | |
| --- | --- | --- | --- | --- | --- | --- | --- |
| **SUMMARY** | | | | | | | |
| *Groups* | *Count* | *Sum* | | *Average* | | *Variance* | |
| Control | 3 | 0.13539 | | 0.04513 | | 0.00001 | |
| EGF | 3 | 0.06408 | | 0.02136 | | 0.00004 | |
| FGF2 | 3 | 0.96492 | | 0.32164 | | 0.00758 | |
| **ANOVA (single-factor)** | | | | | | | |
| *Source of Variation* | *SS* | *df* | | *MS* | *P-value* | | *F crit* |
| Between Groups | 0.16719 | 2 | | 0.08360 | **0.00058*** | | 5.1433 |
| Within Groups | 0.01526 | 6 | | 0.00254 |  | |  |
| Total | 0.18245 | 8 | |  |  | |  |
| **Multiple comparison with Tukey HDS**  Q-critical = 4.34 (Df =6, k=3, significance = 0.05) | | | | | | | |
|  | Control vs. EGF | | Control vs. FGF2 | | EGF vs. FGF2 | | |
| HDS | 0.81642 | | **9.49764*** | | **10.31406*** | | |
| HDS> q-critical | No | | **Yes*** | | **Yes*** | | |
| Adj. p-value  (anova-tukey in R) | 0.8368 | | **0.0013*** | | **0.0008*** | | |

**Supplementary Table 2.**

| **Statistical summary for Figure 1D.** Asterisk indicates statistical significance. | | | | | | | |
| --- | --- | --- | --- | --- | --- | --- | --- |
| **SUMMARY** | | | | | | | |
| *Groups* | *Count* | *Sum* | | *Average* | | *Variance* | |
| Control | 3 | 1.49317 | | 0.49772 | | 0.00968 | |
| EGF | 3 | 0.68749 | | 0.22916 | | 0.00385 | |
| FGF2 | 3 | 0.60166 | | 0.20055 | | 0.00467 | |
| **ANOVA (single-factor)** | | | | | | | |
| *Source of Variation* | *SS* | *df* | | *MS* | *P-value* | | *F crit* |
| Between Groups | 0.16125 | 2 | | 0.08063 | **0.00624*** | | 5.14325 |
| Within Groups | 0.03639 | 6 | | 0.00606 |  | |  |
| Total | 0.19764 | 8 | |  |  | |  |
| **Multiple comparison with Tukey HDS**  Q-critical = 4.34 (Df =6, k=3, significance = 0.05) | | | | | | | |
|  | *Control vs. EGF* | | *Control vs. FGF2* | | *EGF vs. FGF2* | | |
| HDS | **5.97315*** | | **6.60947*** | | 0.63632 | | |
| HDS> q-critical | **Yes*** | | **Yes*** | | no | | |
| Adj. p-value  (anova-tukey in R) | **0.0131*** | | **0.0082*** | | 0.8963 | | |

**Supplementary Table 3.**

| **Statistical summary for Figure 1E.** Asterisk indicates statistical significance. | | | | | | | |
| --- | --- | --- | --- | --- | --- | --- | --- |
| **SUMMARY** | | | | | | | |
| *Groups* | *Count* | *Sum* | | *Average* | | *Variance* | |
| Control | 3 | 0.281071 | | 0.09369 | | 0.000591 | |
| EGF | 3 | 0.278383 | | 0.092794 | | 2.51E-05 | |
| FGF2 | 3 | 4.88074 | | 1.626913 | | 0.01137 | |
| **ANOVA (single-factor)** | | | | | | | |
| *Source of Variation* | *SS* | *df* | | *MS* | *P-value* | | *F crit* |
| Between Groups | 4.704294 | 2 | | 2.352147 | **1.3E-07** | | 5.143253 |
| Within Groups | 0.023972 | 6 | | 0.003995 |  | |  |
| Total | 4.728265 | 8 | |  |  | |  |
| **Multiple comparison with Tukey HDS**  Q-critical = 4.34 (Df =6, k=3, significance = 0.05) | | | | | | | |
|  | Control vs. EGF | | Control vs. FGF2 | | EGF vs. FGF2 | | |
| HDS | 0.0245 | | **42.0138*** | | **42.0383*** | | |
| HDS> Q-critical | no | | **Yes*** | | **Yes*** | | |
| Adj. p-value  (anova-tukey in R) | 0.9998 | | **3.94E-7*** | | **3.93E-7*** | | |

**Supplementary Table 4.**

| **Statistical summary for Figure 2A.** Asterisk indicates statistical significance. | | | | | | | |
| --- | --- | --- | --- | --- | --- | --- | --- |
| **SUMMARY** | | | | | | | |
| *Groups* | *Count* | *Sum* | | *Average* | | *Variance* | |
| Control | 10 | 41723.13 | | 4172.31 | | 2217675.07 | |
| EGF | 7 | 49105.25 | | 7015.04 | | 739248.65 | |
| FGF2 | 10 | 79305.33 | | 7930.53 | | 3993123.19 | |
| **ANOVA (single-factor)** | | | | | | | |
| *Source of Variation* | *SS* | *df* | | *MS* | *P-value* | | *F crit* |
| Between Groups | 75435807 | 2 | | 37717903 | **5.93E-5** | | 3.4028 |
| Within Groups | 60332676 | 24 | | 2513861 |  | |  |
| Total | 135768483 | 26 | |  |  | |  |
| **Multiple comparison with Tukey HDS**  Q-critical = 3.532 (Df =24, k=3, significance = 0.05) | | | | | | | |
|  | *Control vs. EGF* | | *Control vs. FGF2* | | *EGF vs. FGF2* | | |
| HDS | **4.7437*** | | **7.4957*** | | 1.5277 | | |
| HDS> q-critical | **Yes*** | | **Yes*** | | no | | |
| Adj. p-value  (anova-tukey in R) | **0.0036** | | **5.6E-5*** | | 0.4810 | | |

**Supplementary Table 5.**

| **Statistical summary for Figure 2A.** Bold with Asterisk indicates statistical significance for anova, while significant comparisons are listed for post hoc and additional | | | | |
| --- | --- | --- | --- | --- |
|  | *Anova, single-factor, with Dunnetts Post hoc test*  *Used to determine significance reported on graphs* | | |  |
| *Wavenumber*  *( cm ^-1^)* | *p-value- between groups* | *F value*  *(F crit = 3.06)* | *dunnett’s critical value-significant comparisons listed* | *Significant p-values listed*  *2 tailed, Student t-test*  *(0.05, Bonferroni correction)* |
| 569 | **6.55E-08*** | 18.55 | **Control vs. EGF***  **Control vs. FGF2***  **EGF vs. FGF2*** | Control vs. EGF= 7.62E-09  Control vs. FGF2=1.04E-03  EGF vs. FGF2=7.76E-03 |
| 621 | **2.47E-14*** | 39.07 | **Control vs. EGF***  **Control vs. FGF2*** | Control vs. EGF= 4.01E-07  Control vs. FGF2= 5.30E-14  EGF vs. FGF2= 9.81E-03 |
| 675 | **1.29E-05*** | 12.17 | **Control vs. EGF***  **EGF vs. FGF2*** | Control vs. EGF= 8.06E-06  Control vs. FGF2= 1.05E-02  EGF vs. FGF2= 1.42E-02 |
| 1124 | **1.36E-09*** | 23.53 | **Control vs. EGF***  **Control vs. FGF2***  **EGF vs. FGF2*** | Control vs. EGF= 7.08E-03  Control vs. FGF2= 1.00E-09  EGF vs. FGF2= 8.94E-06 |
| 1248 | **5.28E-07*** | 15.97 | **Control vs. FGF2***  **EGF vs. FGF2*** | Control vs. FGF2= 1.66E-07  EGF vs. FGF2= 4.11E-06 |
| 1335 | **8.97E-07*** | 15.33 | **Control vs. FGF2***  **EGF vs. FGF2*** | Control vs. FGF2= 3.53E-07  EGF vs. FGF2= 3.99E-06 |
| 1446 | **2.30E-06*** | 14.20 | **Control vs. FGF2***  **EGF vs. FGF2*** | Control vs. FGF2= 5.40E-07  EGF vs. FGF2= 9.85E-06 |
| 1654 | **5.36E-06*** | 13.20 | **Control vs. FGF2***  **EGF vs. FGF2*** | Control vs. FGF2= 1.58E-03  EGF vs. FGF2= 3.39E-06 |
| 1927 | **3.22E-07*** | 16.58 | **Control vs. FGF2***  **EGF vs. FGF2*** | Control vs. FGF2= 9.73E-08  EGF vs. FGF2= 2.98E-06 |
